# The Educational Treatment of Idiots. Imbeciles, and Feeble-Minded Children

**Published:** 1894-03-24

**Authors:** G. E. Shuttleworth

**Affiliations:** late Medical Superintendent, Royal Albert Asylum, Lancaster; formerly Assistant Medical Officer, Earlswood Asylum


### The Educational Treatment of Idiots, Imbeciles, and Feeble-Minded Children.

By G. E. Shuttleworth, B.A., M.D., &c., late Medical Superintendent, Royal Albert Asylum, Lancaster; formerly Assistant Medical Officer, Earlswood Asylum.

The story of efforts to reclaim our mental waifs and strays belongs, like so many other philanthropic movements, exclusively to the nineteenth century. In the literature of Greece and Rome we find but few references to what we now call the "afflicted classes": the ἰδιώτης of antiquity was but the "private person" who held aloof from politics, the "imbecillus" was (at least according to one etymology) the man who had to walk with a crutch. Of old the natural law of the survival of the fittest was also the law of the nations. Lycurgus ordained that deformed or weakly Spartan infants were to be exposed to perish on Mount Taygetus. Though in Christian times the law of compassion for the weak was gradually evolved, and there is even apostolic authority for "suffering fools gladly," and "comforting the feeble-minded," we find that even as late as the last century idiot children were, in various parts of Christendom, abandoned by their parents to the tender mercies of the denizens of the forest. Linnæus even describes, as a variety of the genus "Homo," some ten of these deserted beings, under such designations as "Juvenis Lupinus Hessensis, 1544" (the Hessian Wolf-youth); "Juvenis Ovinus Hibernicus" (the Irish Sheep-youth); and so on. The history of these cases is obscure, but in 1724 Blumenbach described a well-authenticated instance of an idiot child, who had grown up amongst the *feræ naturæ* of a Hanoverian wood. This lad, afterwards known as "Peter the Wild Boy," was seen in England by Dean Swift, who noted his peculiarities. In 1798 a boy, apparently some twelve years old, was captured by the sportsmen in the forest of Canne, department of Aveyron, France, wild and naked. He had subsisted on roots and acorns. Speechless, and of savage mien, he looked more like a beast than a human being. In Paris he fortunately fell into the hands of M. Itard, Physician to the Deaf Mute Institution, who tried all he knew to reclaim this "simple child of nature," better known, however, under the designation of "le Sauvage de l'Aveyron." After five years' unavailing efforts to educate him, he was forced to concur with his friend Pinel that the lad was but an idiot, and consequently ineducable! In spite of this pessimistic view, Itard's labours were by no means in vain, for his philosophic analysis of the sensory and mental deficiencies of the case, and his ingenuity in adapting educational methods to its necessities, entitle him to be regarded as one of the founders of the system of training subsequently so successful in the amelioration of the imbecile.

Ferret, Fabret, Voisin, Esquirol, and others in Paris seem to have made attempts to improve the mental condition of idiots in the large hospices of that city; but comparatively little was achieved until M. Édouard Séguin was, in 1842, appointed the instructor of imbeciles at the Bicêtre. Here he worked out those principles of "physiological education"—so called in contradistinction to the mere training of memory then in vogue—which, starting with the cultivation of the senses, opened the way to the development of the intelligence of imbeciles in a manner never before dreamed of. Séguin's classic work, entitled "Traitement Moral, Hygiène et Éducation des Idiots," published in 1847, may indeed be regarded as the Magna Charta of the emancipation of this unfortunate class.

Not in Paris alone, however, but also in Switzerland and Germany, movements were started at about the same period for the relief of the feeble in mind. Thus, in 1842, Dr. Guggenbühl established on the Abendberg above Interlaken, at an altitude of 3,000 feet, the first institution for the amelioration of Cretins by hygienic and educational methods. In 1842, also a department for the instruction of idiots in connection with the School for Deaf Mutes at Berlin was opened by Dr. Saegert.

In Great Britain little was done in this direction until interest was aroused by an account, published by Dr. William Twining in 1843, of what he had witnessed on the Abendberg, one result of which was the opening, at Bath, in 1846, by the Misses White, of a small home for idiot children—the first in this country. Knowledge of what had been done abroad—more especially by Séguin—was further advanced by the publication, early in 1847, of articles in *Chambers' Journal* and the *British and Foreign Medico-Chirurgical Review* by Mr. Gaskell and Dr. Conolly respectively. The good seed thus sown brought forth fruit later in the year in the establishment, by the philanthropic exertions of Drs. Andrew Reed, Conolly, and others, of a National Institution for the benefit of Idiots, and Park House, Highgate, was opened in 1848 as the first public asylum for this class in England. Supplementary accommodation was obtained in 1850 at Essex Hall, Colchester; but the two establishments proving inadequate, a building large enough for 500 inmates was erected on a picturesque site at Earlswood, Surrey, the foundation-stone being laid by the late Prince Consort in June, 1853. Subsequently Essex Hall, Colchester, became a separate institution for the Eastern Counties, and in 1864 and 1866 respectively Idiot Asylums for the Western and the Midland Counties were established. In 1864 the project of a large institution, on the model of Earlswood, for the Northern Counties of England, was inaugurated, and as a result the Royal Albert Asylum at Lancaster (planned for 600 inmates) was opened in 1870. In 1875 the first Pauper School for Idiots was opened at Clapton under the Metropolitan Asylums Board, and this ultimately developed into the large establishment at Darenth accommodating 1,000 juvenile imbeciles, besides adults. Blocks for pauper idiots have also been established in connection with the County Lunatic Asylums for Warwick and Northampton, and are now being organised in the counties of Middlesex and Essex. With these few exceptions, however, no systematic provision has been made for pauper idiots in this country, and in the main charity has been left to do the work which would seem to have special claims upon the State as guardian of these poor children—

> Fashioned and shaped by no will of their own,
> And helplessly into life's history thrown;
> Born by the law that compels men to be,
> Born to conditions they cannot foresee.

